# Clinical Characteristics in Patients with Rheumatoid Arthritis: Differences between Genders

**DOI:** 10.1155/2019/8103812

**Published:** 2019-07-03

**Authors:** M. Intriago, G. Maldonado, J. Cárdenas, C. Ríos

**Affiliations:** ^1^Universidad Espíritu Santo, Km. 2.5 Vía la Puntilla Samborondón, Ecuador; ^2^Centro de Reumatología y Rehabilitación, El Oro y Ambato 1004, Guayaquil, Ecuador

## Abstract

**Objective:**

To compare the clinical characteristics of a group of men and women with rheumatoid arthritis (RA) and determine the differences between genders.

**Materials and Methods:**

A descriptive and comparative cross-sectional study was developed with a group of 50 men and a control group of 50 women with RA, from a rheumatology center in the city of Guayaquil, Ecuador. Data collected included clinical manifestations, comorbidities, treatment, and disease activity. Clinical and activity differences between sexes were analyzed.

**Results:**

Women were more devoted to housework (66%), while men consumed more tobacco (34%) and alcohol (38%). Fatigue (60%), loss of appetite (54%), and weight loss (44%) were more common in women. No differences were found in comorbidities or treatment. Women had higher values of DAS-28 (3.4 vs 2.5), HAQ-DI (1.1 vs 0.4), ESR (33.0 vs 23.2), painful joints (8 vs 3), swollen joints (6 vs 2), and overall physician assessment (3 vs 2).

**Conclusion:**

The results are similar to other publications that establish that women have a more aggressive disease with greater activity of the disease and disability.

## 1. Introduction

Rheumatoid arthritis (RA) is a systemic inflammatory disease that is characterized by a progressive and disabling course [1]. The prevalence of RA is 0.5-1%, with a woman to man ratio of 3:1 [2]. It is 4 to 5 times higher in women under 50 years, but after 60 years the ratio becomes approximately 2 to 1 [3]. In Latin America, the prevalence of this disease is high with a ratio of 5.2 women per man in Colombia [4], 5.2 to 1 in Argentina [5], and 5.5 to 1 in Cuba [6].

When studying the influence of gender on the clinical course of the disease, the results have been contradictory. Some studies report a less favorable state in men [7], while other authors claim the opposite [8–11].

Lesuis et al. [12], Sokka et al. [13], and Hallert et al. [14] have shown that women have greater functional disability, disease activity, and pain than men. It has also been found that being a man is a predictor of remission [8, 9] and that women have greater work disability in terms of loss of work days and productivity [15]. The aim of this study is to describe and compare the clinical and serological characteristics of a group of men with RA compared to a control group of women.

## 2. Materials and Methods

A cross-sectional study was carried out in patients with preestablished diagnosis of RA from a rheumatology center in the city of Guayaquil. The time period was 6 months from June 2016 to December of the same year.

### 2.1. Characteristics of the Population

A group of 50 men and a control group of 50 women were randomly chosen. Only patients over 18 years of age were included. Patients with other connective tissue diseases and those who did not wish to participate in the study were excluded. After informed consent was obtained, information was collected about demographic data, habits, clinical manifestations, comorbidities, treatment, painful and swollen joint count, visual analogue scale for pain (VAS), and physician assessment. Alcohol consumption was defined as consuming 15 drinks or more per week in men and 8 drinks or more per week in women. A smoker was defined as a person who currently smokes cigarettes or who has smoked 100 cigarettes in his lifetime.

In addition, patients filled the Health Assessment Questionnaire Disability Index (HAQ-DI) to assess functional capacity and the Patient Health Questionnaire-9 (PHQ-9) for depression.

The laboratory data from the patient's last clinical history was accessed, from which we obtained C-reactive protein (CRP), erythrocyte sedimentation rate (ESR), rheumatoid factor (RF), and anti-Cyclic Citrullinated Peptide (anti-CCP). With these data, the DAS-28 disease activity index was calculated.

According to the latest statistics from the National Institute of Statistics and Census (INEC) [16], the population of Ecuador exceeds 17 million inhabitants, with 50.4% being women, with an index of 98.2 men for every 100 women. 71.9% of the population define themselves as mestizos. 62.8% live in urban areas. The unemployment rate in the Ecuadorian population is 4.4%, adequate employment 41.1%, and underemployment 18.3%. As for the employees, 58.7% are men and 41.3% are women. As for the unemployed, 48.2% are men and 51.8% are women. Illiteracy in women is about 7.7%, 1.9 percentage points higher than male illiteracy. More than 900,000 Ecuadorians consume alcohol: 89.7% are men and 10.3% are women; 431 500 Ecuadorians smoke: 85.5% are men and 14.5% are women. The prevalence of RA in Ecuador is 0.9%.

### 2.2. Statistical Analysis

The statistical program SPSS V. 22 was used to analyze the data and calculate frequencies, percentages, means, standard deviations, minimum, and maximum. To compare the ordinal data, the chi square test was used, and to compare means, the ANOVA coefficient. The statistical significance used was 0.05 with a reliability of 95%.

## 3. Results

The mean age of the men was 49 years and of women 47 years. The majority of the patients were mestizos. Regarding marital status, 76% of men and 60% of women were married. The majority came from urban areas, 90% men and 86% women. 72% of the men worked while 66% of the women performed housework (p<0.05). Likewise, smoking was more common in men (34% vs 8% p <0.05), as was alcohol consumption (30% vs 0% p <0.05).  Table 1 shows the demographic characteristics of both groups.

Regarding the characteristics of the disease, the average age of onset was 40 years for both groups. There was no significant difference in the delay until the visit with the specialist: 24 ±57 months in men and 32± 46 months in women. The rheumatoid factor was positive in 90% of men and 96% women, and anti-CCP in 84% men and 86% women.

**Figure 1:**
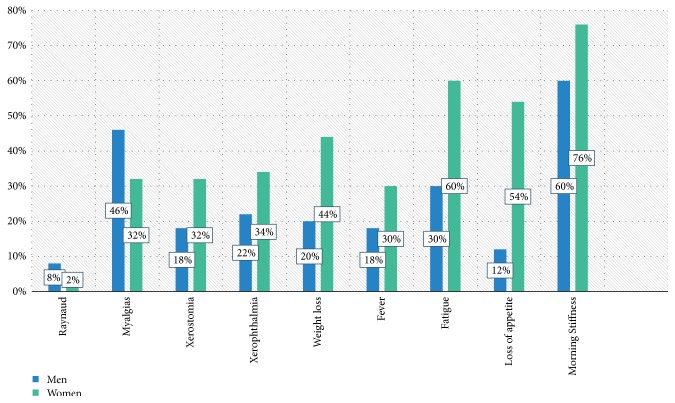
Clinical manifestations.

The disease began in the majority of patients insidiously, 56% men and 52% women, with symmetrical involvement of 82% men and 74% women. Regarding extra-articular manifestations (Figure 1), women had greater fatigue (60% vs. 30% p=0.003), weight loss (44% vs. 20% p=0.010), and loss of appetite (54% vs. 12% p≤0.001).

**Figure 2:**
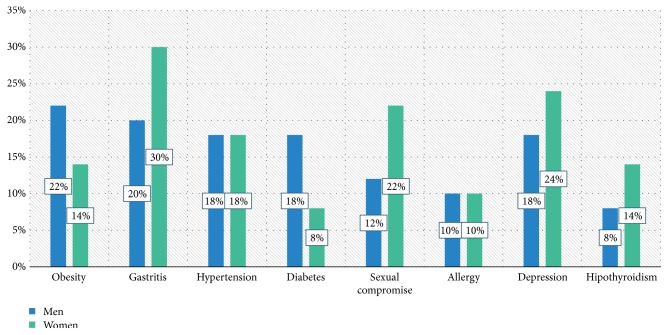
Comorbidities.

**Figure 3:**
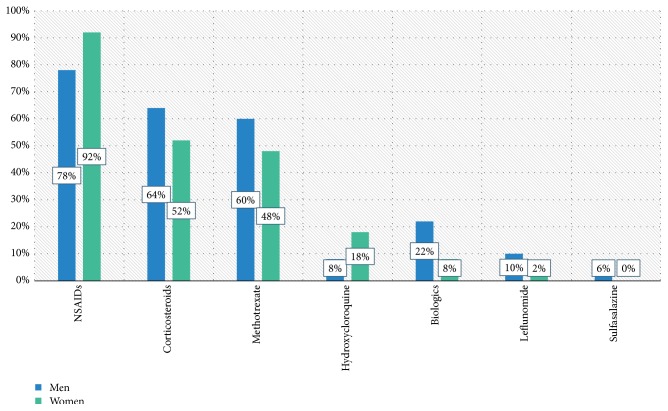
Treatment.

There were no significant differences in comorbidities (Figure 2) or in the treatment (Figure 3).

Men showed less disease activity in terms of physician's assessment (2 in men vs 3 in women), painful joint count (3 in men vs 8 in women), swollen joint count (2 in men vs 6 in women), ESR (23.2 mm/h in men vs 33.0 mm/h in women), DAS-28 (2.5 in men vs 3.4 in women), and HAQ-DI (0.37 in men vs 1.12 in women) (p <0.05) (Table 2). Likewise, the prevalence of functional disability and severe disability was lower in the group of men. No significant differences were found in VAS of pain (3 vs 4), CRP (13 mg / L vs 29 mg / L), or PHQ-9 (5 vs 4).

## 4. Discussion

When comparing the group of men with RA with the control group of women, significant differences were found regarding habits, clinical manifestations, and disease activity.

Smoking was more common in men, as in the study by Krishnan, Sokka, and Hannonen [17]. Voulgari et al. [18] found no difference in weight loss between genders while in this study women had greater weight loss. The prevalence of fatigue was higher in women, similar to the study by Sokka et al. [13]. On the other hand, there was no difference in the presence of dry symptoms, unlike the study by Weyand et al. [7].

In the study by Hallert et al. [14] 33% of patients presented some comorbidity, without differences between sexes. In the present study, no differences were found regarding comorbidities. In contrast, Albrecht [19] found that depression, fibromyalgia, and hypothyroidism were more frequent in women while cardiovascular diseases and diabetes were more frequent in men.

Similar to this study, Sokka et al. [13] found that measures of disease activity were higher in women than in men, including swollen joint count, tender joint count, ESR, VAS for physician global estimate, pain, and patient global status. Other studies have found higher DAS28 remission rates [8, 9] and treatment responses in men [11].

van Vollenhoven [20] raises concern as to whether these differences between genders are due to an inherent difference in the biology of the disease, differences in treatment or response to the same treatment between men and women, subjective experience of the disease, or measurements of disease that are not sex neutral. Likewise, Sokka et al. [13] suggested that most of the gender differences can originate from the measures of disease activity instead of the activity of the disease itself, since they found that among patients who had 0 to 1 swollen joint, women had higher values than men for all other subjective disease activity measures.

Women have higher ESR [21] and worse scores [22] in most of the questionnaires; therefore, the activity indexes for this group are usually worse. Leeb et al. [23] showed that the DAS28 values differed considerably according to sex and the perception of pain. Women are more likely to experience different types of recurrent pain and report higher scores [24]. Furthermore, it has been found that the pain threshold is lower in women and that they experience more physical pain than men for the same noxious stimulus [25].

When measuring disease severity in terms of structural damage, Gossec et al. [26] did not find differences in radiographic scores between genders. Another study [27] also found similar percentages of erosive disease in men and women.

It has been reported that female sex is a predictor of disability and that the progression of disability is three times faster in women [28]. In the present study, women had a higher HAQ-DI score and a higher prevalence of disability, as in the study by Häkkinen et al. [29], in which these differences were attributed to differences in muscle strength and pain score. As women have a lower muscular strength than men, the impact of RA in the functional capacity is greater in this group.

Sex hormones also play an important role in the differences between genders. The severity of RA correlates inversely with androgen levels, which is a possible explanation for the lower severity of the disease in men [30]. Testosterone interacts with the immune system suppressing the humoral and cellular response [31]. Other possible explanation according to Lesuis et al. [12] is that there is a possible subtreatment and a longer duration of the disease in women. Lard et al. [32] also reported a longer delay in treatment in women with early arthritis compared to men. We did not find any differences in treatment or delay until the visit to the specialist.

In this study, women presented with clinical characteristics and measures of disease activity more severe than men, which agrees with previous publications. It is not completely clear whether these differences are due to the intrinsic nature of the disease or to the instruments utilized to assess the disease severity. Also, different gender coping mechanisms can influence this outcome. Since sex differences influence therapeutic goals and response to treatment, these must be considered in the individual therapeutic approach of each patient. In Ecuador there are no other studies comparing the characteristics of RA between men and women; however, there is another descriptive study of patients with RA that included 353 women and 37 men, with similar epidemiological characteristics [33].

## 5. Conclusion

It is evident that women have greater activity of the disease than men, represented in poorer quality of life and associated comorbidities such as depression. Due to this, it is important to implement screening tests and perform multidisciplinary and integral management. When considering gender differences, one has to take into account the fact that disease activity measures themselves may be influenced by gender.

## Figures and Tables

**Table 1:** Demographics.

	Men (N = 50)	Women (N = 50)	p
Mean age	49 ± 13	47 ± 13	N.S

*Ethnicity*			

White	1 (2%)	1 (2%)	N.S

Mestizo	47 (94%)	49 (98%)	N.S

Afro-Ecuadorian	2 (4%)	-	N.S

*Marital status*			

Single	6 (12%)	1 (2%)	N.S

Married	38 (76%)	35 (70%)	N.S

Divorced	5 (10%)	10 (20%)	N.S

Widow	1 (2%)	4 (8%)	N.S

*Area*			

Urban	45 (90%)	43 (86%)	N.S

Rural	5 (10%)	7 (14%)	N.S

*Occupation *			

Work	36 (72%)	15 (30%)	≤0.001

No work	14 (28%)	2 (4%)	≤0.001

Household chores	-	33 (66%)	≤0.001

*Habits*			

Smoking	17 (34%)	4 (8%)	≤0.001

Alcohol	19 (38%)	-	≤0.001

N.S: nonsignificant.

**Table 2:** Markers of disease activity.

	Men (N = 50)	Women (N = 50)	p
Physician assessment	2 [0-8]	3 [0-9]	0.047

VAS pain	3 [0-8]	4 [0-9]	N.S

Painful joint count	3 [0-24]	8 [0-28]	≤0.001

Swollen joint count	2 [0-12]	6 [0-26]	≤0.001

ESR (mm/h)	23.2 [1-68]	33.0 [19-70]	0.046

CRP (mg/L)	13.0 [2-57]	29.4 [1-112]	N.S

PHQ-9	5 [0-19]	4 [0-17]	N.S

DAS-28	2.5 [0, 9-5, 8]	3.4 [0, 9-7]	0.011

(i) Remission	27 (54%)	19 (38%)	NS

(ii) Low activity	8 (16%)	6 (12%)	NS

(iii) Moderate Activity	14 (28%)	13 (26%)	NS

(iv) High activity	1 (2%)	12 (24%)	≤0.001

HAQ-DI	0.37 [0-3]	1.12 [0-3]	≤0.001

(i) Functional disability	6 (12%)	18 (36%)	0.005

(ii) Severe disability	1 (2%)	11 (22%)	0.002

N.S: nonsignificant.

## Data Availability

The data used to support the findings of this study are included within the article.
